# CLOCK Genes and Circadian Rhythmicity in Alzheimer Disease

**DOI:** 10.4061/2011/383091

**Published:** 2011-10-18

**Authors:** J. Thome, A. N. Coogan, A. G. Woods, C. C. Darie, F. Häßler

**Affiliations:** ^1^Department of Psychiatry, University of Rostock, Gehlsheimerstraße 20, 18147 Rostock, Germany; ^2^Department of Psychology, National University of Ireland, Maynooth, Maynooth, Ireland; ^3^Biochemistry and Proteomics Group, Department of Chemistry and Biomolecular Science, Clarkson University, Potsdam, NY 13699, USA; ^4^Department of Child and Adolescent Psychiatry and Neurology, University of Rostock, 18147 Rostock, Germany

## Abstract

Disturbed circadian rhythms with sleep problems and disrupted diurnal activity are often seen in patients suffering from Alzheimer disease (AD). Both endogenous CLOCK genes and external Zeitgeber are responsible for the maintenance of circadian rhythmicity in humans. Therefore, modifications of the internal CLOCK system and its interactions with exogenous factors might constitute the neurobiological basis for clinically observed disruptions in rhythmicity, which often have grave consequences for the quality of life of patients and their caregivers. Presently, more and more data are emerging demonstrating how alterations of the CLOCK gene system might contribute to the pathophysiology of AD and other forms of dementia. At the same time, the impact of neuropsychiatric medication on CLOCK gene expression is under investigation.

## 1. Introduction

Alzheimer disease (AD) is the most frequent form of dementia and one of the most devastating psychiatric disorders, with an estimated 33.9 million people worldwide affected [1]. With increasing life expectancy, AD prevalence will further rise dramatically within the next decades. While the typical neuropathological features have been well known since their first description in 1906 [2], the complex pathophysiology of this condition is still not fully understood. Some progress has been made in elucidating possible risk genes [3, 4] as well as the role of advanced glycation end products [5, 6], amyloid plaques [7], neurofibrillary tangles [8], and other pathological factors which probably interact within the so-called “pathogenic cascade” [9]. Further, presently available treatment strategies are not sufficient and not satisfying as they, at best, slow down the progression of the symptoms but do not provide an option to reverse them. Intervention may frequently occur at advanced stages of neurodegeneration which are no longer possible to alleviate [10]. Additionally, treatments are often associated with a plethora of side effects. 

From a clinical point of view, patients, their families, carers, and health professionals are confronted with severe challenges in managing the condition. One such typical challenge is the disturbed diurnal-nocturnal rhythm of many Alzheimer patients [11–13]. Interestingly, circadian CLOCK gene polymorphisms might be linked to sleep-wake disturbances in Alzheimer patients, although the authors of a recent paper focusing on this hypothesis reported largely negative findings for the 122 circadian-related polymorphisms included [14]. The molecular and cellular basis of circadian rhythmicity consists of a complex interaction between various transcription factors, commonly described as CLOCK genes, which represent an internal timekeeping system that interacts with external synchronising factors: the so-called Zeitgebers [15, 16]. Via this interaction, humans and many animals maintain a recurring 24-hour (circadian) system for a plethora of biological and behavioural processes. Desynchronisation can be caused by various factors such as shiftwork, airtravelling through time zones, substances and medication, and neuropsychiatric disorders [17, 18].

Severe desynchronisation is regularly observed in patients suffering from AD and, therefore, the elucidation of the role of CLOCK genes in this condition might contribute to a significantly improved understanding of its underlying pathomechanisms.

## 2. Sleep and Circadian Rhythmicity in Alzheimer Patients

Nocturnal sleep disturbance is often reported by caregivers of Alzheimer patients and seems to be associated with disease severity [19]. Additionally, sleep quality is often very poor [20] and AD patients suffering from apathy seem to exhibit less consolidated nocturnal sleep [21]. Furthermore, disturbed sleep patterns in patients can have adverse consequences for the health of caregivers [22].

Interestingly, a delayed endogenous circadian phase of core body temperature was found in patients with probable AD by Harper and coworkers [13]. The same group also described that the deviation from normal circadian rhythmicity increases with the severity of Alzheimer pathology [23] and that there might be distinct differences in the pattern of desynchronisation depending on the type of neurodegeneration [12]. These findings can be interpreted as support for the hypothesis that disturbed circadian rhythmicity in patients with AD is mediated by pathological CNS (central nervous system) changes.

Hatfield et al. [23] monitored rhythmicity via actigraphy and cortisol determination in a group of mildly and moderately demented Alzheimer patients over the course of a year and report that as disease progresses, so does the activity rhythm, but the cortisol rhythm is relatively spared. These findings suggest that in the earlier stages of the disease process, circadian deterioration may not be a global phenomenon, but may affect specific domains. A recent study by Hu et al. [24] shows that AD attenuates the scale invariance of the motor rhythm, perhaps reflecting pathological alterations in the SCN (suprachiasmatic nucleus). Such pathological changes in the SCN have been noted in postmortem analysis of the brains from severely demented patients, with neuronal loss and tangles noted, but rarely plaques in the SCN [25]. There are also decreases in specific neurochemical phenotypes in the SCN that are lost [25, 26], with such neurochemicals (VIP (vasoactive intestinal peptide), AVP (arginine-vasopressin)) being crucially implicated in SCN function. Such disturbances in SCN function and the resultant circadian abnormalities are postulated to in part underpin “sundowning” in Alzheimer patients, in which behavioural agitation arises in the late afternoon/evening [27]. In this context, the results of an animal model experiment are interesting: the study involved the implantation of genetically modified, beta/A4-expressing cells into the SCN of adult rats. It was possible to demonstrate that such overexpression of beta/A4 amyloid led to significant alterations in the activity pattern of the animals and disruption of circadian rhythmicity [28]. The 3xTg (triple transgenic) mouse models of AD, which exhibits both Abeta and tau neuropathology, show deteriorated circadian organisation of locomotor behaviour, although there was no weakening of the ability of the system to respond appropriately to light stimulus, whilst the SCN of these animals also showed a decrease of VIP and AVP cells [29]. Bero et al. [30] demonstrated, again in a murine model, that the concentration of interstitial-fluid Abeta and, thus, the Abeta aggregation depend on endogenous neuronal activity, implying that disturbed sleep could contribute to amyloid deposition. Craig et al. [31] suggested that the common and distressing sleep disruption in Alzheimer patients is associated with genetic factors, specifically with a variation of the MAO-A (monoamine oxidase A) gene. Other obvious candidate genes are CLOCK genes. While this gene family has been implicated in other neuropsychiatric disorders [32], a recent study failed to reveal possible associations between CLOCK gene single-nucleotide polymorphisms (SNPs) and sleep-wake disturbance in AD [14]. Nevertheless, even if CLOCK gene variants have not yet been identified as possible risk factors, it is possible that the expression of these genes is altered in Alzheimer patients and that a changed expression pattern of these genes contributes to disturbed circadian rhythms [33].

## 3. The Role of CLOCK Genes in AD

It has been shown that age, the most important risk factor for AD, influences the expression of molecules involved in immunity as well as in cell signaling and transcription factor regulation, such as transforming growth factor-beta and phosphorylated SMAD3 (Mothers against decapentaplegic homolog 3), in the mouse suprachiasmatic (SCN) and paraventricular nuclei, that is, the neuroanatomical areas crucially involved in the maintenance of circadian rhythmicity [34].

In a recent postmortem study, Cermakian and coworkers found significant differences in the expression pattern of CLOCK genes between Alzheimer patients and controls. The RNA (ribonucleic acid) expression level of the CLOCK gene PER1 (period circadian protein homolog 1), PER2 (period circadian protein homolog 2), and BMAL1 (brain and muscle aryl-hydrocarbon-receptor-nuclear translocator- (ARNT-) like) were quantified via RT-PCR (reverse transcription polymerase chain reaction) for the following brain areas: bed nucleus of the stria terminalis, cingulate cortex, and the pineal gland [33]. A previous study had already shown that in the brain of Alzheimer patients vasopressin gene expression is altered in the SCN [35].

Additionally, a possible noncircadian role of CLOCK genes in psychiatric disorders has been discussed, mainly affecting behavioural phenomena; however, this could also be an epiphenomenon of underlying rhythmicity at the cellular and systemic level [36].

A further important point is also the possibility of using a “resetting” of the internal clock in the treatment of AD. For example, there is evidence that the use of light therapy may delay cognitive decline in AD as well as noncognitive symptoms such as depression [37, 38].

In this context, the possible impact of AD medication on CLOCK gene expression and, thus, indirectly on circadian rhythmicity must also be discussed. To our knowledge, there are no studies published which address this issue specifically for medication used in Alzheimer treatment. However, there is considerable evidence that neuropsychiatric medication can significantly alter the expression of CLOCK genes in various brain areas. In animal models, haloperidol alters the circadian CLOCK gene expression in the murine CNS [17]. Further, medication with typical neuroleptic haloperidol has been found to alter the rhythmicity of rest-activity cycle and to deteriorate the cognitive state in early-onset Alzheimer patients [39].

## 4. The Dilemma of How and When to Monitor the CLOCK Genes

 There are significant difficulties in monitoring CLOCK genes, either when one monitors the genes themselves or their RNA or protein products. Starting at the gene level, it is important to know exactly when the material was collected during the day from patients with AD. Since the genes are influenced by circadian rhythms, a sample collected during the night will be different from ones collected during the day (or even morning/evening). Finding a matched control may also be difficult. Background substraction and data manipulation to eliminate false positive or false negative results is also important and difficult to address. It is also important to know what is influenced by circadian rhythms: the gene, the RNA, the protein, protein posttranslational modifications, protein-protein interactions, or a combination of more than one? Also, if only CLOCK proteins are responsible for the circadian phase, what phenomenon affects the level of the proteins per se? Is it the increase in RNA translation (and increased gene transcription) leading to increased protein expression or is it just prevention of protein degradation? Is it reduced rate of phosphorylation or increased rate of dephosphorylation? Trying to answer these and other questions as well as finding the right way to investigate these genes and their transcriptional and translational products, as well as posttranslational modifications and protein-protein interactions is challenging and time consuming.

## 5. Discussion

During recent years, better understanding of the molecular and cellular basis of the clinically frequently observed sleep disturbances and disrupted diurnal and nocturnal rhythms of Alzheimer patients is slowly evolving. CLOCK genes are crucially involved in the maintenance of such rhythmicity, and studies linking this molecule family to brain and body processes in Alzheimer patients can further elucidate the etiopathogenesis of this most frequent dementia. However, to date, the number of such studies is limited and there are only very few reliable and reproducible data available. Further research is urgently needed, especially regarding the exact role CLOCK genes play within the AD pathological cascade and regarding the exact interaction with other pathogenic factors such as amyloid, neurofibrillary tangles, oxidative stress, advanced glycation end products, lipidomic alterations, altered immune modulation, excitotoxicity, and so forth.

Another important feature of CLOCK genes is their close interrelation with external, that is, environmental factors; thus, very few other gene families exhibit such strong gene-environment interaction and interdependency. Therefore, they might be crucial in the integration of exogenous factors and noxae (such as stress) and endogenous vulnerability factors (such as inherited genetic variants), ultimately leading to the clinical phenomenology of AD.

Finally, using and applying this knowledge for the development of improved treatment strategies with better efficacy and tolerability will be an important challenge which, if faced, might lead to significant contributions to the improvement of the quality of life of millions of people suffering from AD.
